# Tracts on Inoculation, Written and Published at St. Petersburg in the Year 1768, by Command of Her Imperial Majesty the Empress of All the Russias: With Additional Observations on Epidemic Small Pox, on the Nature of That Disease, and on the Different Success of the Various Modes of Inoculation

**Published:** 1781-09

**Authors:** 


### THE LONDON MEDICAL JOURNAL,

### For SEPTEMBER 1781.

### SECTION I.

### BOOKS.

I. *Tracts on Inoculation, written and published at St. Petersburg in the year* 1768, *by command of her imperial majesty the empress of all the Russias: with additional observations on epidemic small pox, on the nature of that disease, and on the different success of the various modes of inoculation. By the hon. Baron* T. Dimsdale, *physian and actual counsellor of state to her imperial majesty the empress of all the Russias, and F. R. S.* 8vo. Owen. *London* 1781. 249 pages.

THIS work is divided into seven chapters. In the first of these we have an account of the author's journey into Russia, and of the introduction of inoculation into that country.

It feems that St. Peterfburg, notwithftanding the greateft precautions are taken, is fcarcely ever free from the fmall pox, which proves extremely deftructive. The fatality of the difeafe in the cafe of a beautiful young lady belonging to the court alarmed the emprefs for her own and her fon's fafety, and induced her to fend to England for a proper perfon who might inoculate herfelf and the grand duke, and introduce that falutary practice among her fubjects.

In July 1768 our author was applied to for this purpofe by Mr. Poufchin, the Ruffian minifter, and fat out foon afterwards for St. Peterfburg, accompanied by his fon. On the day after his arrival he waited on the minifter, Count Panin, who with great good fenfe pointed out to him the importance of the office he was called to perform, and begged of him to weigh it properly before he engaged in it. He recommended it to him to be with her majefty and the grand duke as much as poffible, in order to make his obfervations, and to ftudy their conftitutions, and concluded with this falutary admonition: "Let us not be too precipitate; but when every "circumftance has been duly attended to, re-"port your opinion freely, and depend on this, "that if you fhould deem the operation hazard-"ous and advife againft it, we fhall think our-

"felves

"selves equally obliged to you, nor will the ac-
"knowledgments, on account of this expedi-
"tion, be inferior to what it will be upon the
"utmost success."——Soon after this our author and his son were introduced to the empress and grand duke, with each of whom they had the honour to dine.

Although our author was prepared to expect much from the excellent understanding and politeness of her majesty, yet her extreme penetration, and the propriety of the questions she asked concerning the practice and success of inoculation greatly surprized him. In a conversation with this princess relative to her intended inoculation, he requested the assistance of the court physicians; but to this she objected: "It is im-

"possible," said she, "that my physicians can

"have much skill in this operation—their inter-

"position may only tend to embarrass you—my

"life is my own, and I shall with the utmost

"confidence rely on your care alone.—With re-

"gard to my constitution, you could receive no

"information from them.—I have had so good

"a share of health that their advice has never

"been required." Upon his proposing that some experiments might first be made by inoculating some of her own sex, and age, and as near as could be similar habit; the empress replied,

" that if the practice had been novel, or the " least doubt of the general succefs had remain- " ed, that precaution might be neceffary; but " as fhe was well fatisfied in both particulars, " there would be no occafion for delay on any " account."

Two young cadets, who were fuppofed not to have had the fmall pox, were firft inoculated by the author's fon. As every body was anxious for the fuccefs of this firft attempt, a journal of every material appearance was tranfmitted to the author twice a day, and tranflated for the emprefs's perufal. Thefe reports were fo unfavourable that on the fixth day he had determined to go himfelf to affift in taking care of thefe patients, but he was firft commanded to attend on the emprefs. The author mentions her converfation at this interview: " She received me," fays he, " in fo engaging a manner, and ani- " mated me fo much by the encouragement fhe " gave me, that I cannot forbear to relate what " paffed on this occafion. She faid, ' I do not ' like to fee you fo unhappy, tell me what is ' the matter.' I anfwered, ' that the unfavour- ' able accounts received of the patients at Wolf ' Houfe, diftreffed me greatly. I am forry for it ' too,' fhe replied; ' but tell me truly, are you ' cer-

'certain that the cadet's fever is not occafioned

'by inoculation? Moft affuredly not. Then,'

faid fhe, 'difmifs your fears—whatever may be

'the event, it fhall not alter my refolution—

'you fhall perform the operation on me, and

'my example will tend to re-eftablifh the

'practice.'——The evening's report was more favourable: two or three puftules appeared on one of the patients, and the other arm remained well, with fuch an appearance as indicated his having had the fmall pox before.

While his fon was employed with the two cadets, our author was making himfelf acquainted with the conftitutions of his illuftrious patients, and for that purpofe paid his duty to the emprefs once or twice every day, generally dining and paffing great part of the day with the grand duke. At length the time for the inoculation of both was finally determined on. That of the grand duke was publicly talked of, but as nothing was faid refpecting the emprefs's inoculation, every one believed that fhe had laid afide all thoughts of it.

Matters were in this fituation when our author went to affift in the inoculation of four more cadets. The natural fmall pox in a fuitable ftate for the purpofe was difcovered in the fuburbs of the the city. An opinion prevails in Ruſſia, that altho' the operation may be ſalutary to the inoculated, yet it produces certain death to the perſon from whom the matter is taken. For this reaſon the family received him with horror in their countenances. He found the patient with a favourable kind of ſmall pox, gaſping for breath, from the very great heat of the room, it being the commonly received idea, that it is impoſſible to keep ſuch patients too hot. After ſome arguments and the interference of her huſband, the mother of the child conſented that the matter ſhould be taken.

The progreſs of the infection in theſe new patients was by no means favourable. The appearance on the arms was different from what our author had ever experienced; for on the punctured part almoſt immediately aroſe a pimple, which ſoon became one large puſtule, filled with matter, very much reſembling the ſmall pox, completely maturated. This continued to the 7th or 8th day, when the eruptive ſymptoms might in the common courſe have been expected. This ſecond experiment, therefore, turned out wholly ineffectual, as the wounds dried up, and the patients continued in perfect health. "Happily," remarks the author, "I had the good "for-

" fortune to be employed in the ſervice of a prin-

" ceſs, whoſe ſuperior underſtanding and forti-

" tude had prepared her for every event."——

In a memorial preſented to the empreſs he freely acknowledged, that the whole proceſs had been conducted according to his own directions, and that he could account for its failure in no other manner than by ſuppoſing, that all of them had really paſſed through the natural ſmall pox in ſome early period of their lives; and, in order to be ſatisfied in this point, he propoſed that they ſhould be inoculated a ſecond time, in the old manner, by a long inciſion, as practiſed by Dr. Schulenius, a phyſician at St. Peterſburgh, who had aſſiſted in theſe experiments. This propoſal being approved by the empreſs, was carried into execution, and the reſult was, that not the leaſt ſymptom of infection was produced: but her imperial majeſty being already fixed in her reſolution did not wait the iſſue of this ſecond trial, but was inoculated privately in each arm on the 12th of October. This important proceſs and the inoculation of the grand duke are the ſubjects of two diſtinct tracts.——

On the 14th of October certain ſigns of infection appeared in the places of inciſion. On the 18th ſhe was attacked with ſymptoms of the erruptive fever, fever, which continued till the 21ſt, when ſome puſtules appeared on her face and arms. On the 23d ſhe complained of a ſoreneſs in her throat, which was occaſioned by a large puſtule on the upper part of the right tonſil. On the 28th of the ſame month ſhe returned from Czars-coe Selo to St. Peterſburgh in perfect health.

The inoculation of the grand duke, which had been poſtponed for a ſhort time, on account of his being ſeized with the chicken pox, was now reſumed. In a memorial, reprinted in this collection, the author gives his remarks on the conſtitution of his imperial highneſs (who appears to have been of a delicate habit, with glandular ſwellings in his throat and one of his cheeks) and the method of conducting the inoculation, which was performed on the 30th of October. The ſymptoms of eruptive fever came on November 6, and on the 9th puſtules were diſcovered on the ſkin and back. His highneſs was incommoded with a ſore throat from a large puſtule above the *velum pendulum palati*; but the whole number of puſtules did not exceed forty, and on the 22d of November he was perfectly recovered.

Immediately after the recovery of the grand duke our author was informed of the honourable and and generous manner in which her imperial majesty propofed to reward his fervices. He was created a baron of the Ruffian empire, with an annuity for life of five hundred pounds, to be paid him in England, befides ten thoufand pounds fterling, which he immediately received, together with the miniature pictures of the emprefs and grand duke. To this muft be added a thoufand pounds paid to him by M. Poufchin previous to his departure from London, towards defraying the expences of his journey. His fon was likewife honoured with the fame title of baron, and prefented with a gold fnuff-box richly fet with diamonds.

The example of thefe illuftrious perfonages had fuch immediate influence that moft of the nobility were impatient to have their families inoculated. For this purpofe our author and his fon were engaged to vifit Mofcow. In order to be certain of a fupply of matter they took with them a child, whom they inoculated previous to their departure from St. Peterfburgh. As they experienced fome delay in their journey, the eruption of the fmall pox appeared while they were on the road. After being engaged in inoculation for two months, Baron Dimfdale propofed returning to St. Peterfburgh, but was detained ſome time by a dangerous illneſs, which reduced him greatly.

Juſt as he was ſetting out from Ruſſia, on his return to England, he was called to the empreſs, who was attacked with a pleuretic fever, and wiſhed to have his aſſiſtance. He therefore again took up his reſidence in the palace. The ſtate of her pulſe indicated bleeding, and eight ounces of blood were directed to be taken away, but Monſieur Rouſſelin, her ſurgeon, refuſed to bleed her, repreſenting that as ſhe was then in a ſweat, the taking away blood would interrupt the perſpiration, and be attended with danger. This is not the only inſtance of oppoſition which our author appears to have experienced from the medical gentlemen at the court of St. Peterſburgh. His advice, however, was followed, the empreſs experienced immediate relief, and in about three weeks was perfectly recovered. After taking his leave, and receiving freſh proofs of the munificence of her imperial majeſty, Baron Dimſdale was attended to Riga by an officer commiſſioned to ſee that every neceſſary accommodation ſhould be provided for him in the ſame manner as at his firſt arrival in the country.

After this narrative follows a ſhort account of the regulations in the medical college of St. Peterſburgh in 1768, from which it appears that every every phyfician and furgeon muft undergo an examination by the college before he can have liberty to practife in the empire. Until fuch liberty is announced in the public papers, he can have no medicines from the imperial fhop, nor dare any apothecary receive his prefcription or fupply him with medicines. Formerly there was no other than the imperial fhop, the whole expenditure and receipt of which is vefted in the crown; but by degrees it has been found convenient to permit the eftablifhment of free laboratories and apothecaries fhops at St. Peterfburgh and Mofcow, and they begin alfo to be eftablifhed in other principal cities of the empire; but all thofe perfons who have the privilege to keep them, produce fatisfactory proofs of their ability and integrity, and comply with certain requifitions before they are licenfed. In all thefe fhops the prices of medicines are regulated by the college.

The next tract is intituled, "A Defcription "of the method propofed for extending the falu"tary practice of Inoculation through the Ruf"fian empire."——The author recommends a general inoculation in every town or village once in five or fix years; the providing a feparate place of abode for fuch as are deemed improper objects, and a proper houfe and other conveniences for thofe who may chance to have the difeafe feverely. He cautions his readers againft illiterate and ignorant inoculators, and mentions feveral inftances of mal-practice. Amongft others he fpeaks of a man, who, upon the credit of having been his coachman, commenced inoculator, and did fome mifchief; and of another perfon, a farmer near Hertford, who inoculated a great number of perfons, and gave out, that the inoculated fmall pox was not catching; but the whole neighbourhood became infected and many died.

This paper is followed by a fhort eftimate of the number of thofe who die of the natural fmall pox, founded chiefly on the London bills of mortality; from which he concludes, that in general the fmall pox carries off the 8th part of thofe who die in London under two years old. He is credibly informed, that in Ruffia one half die of all that are attacked with this difeafe, the ravages of which in that empire he computes at two millions annually. His mind was deeply impreffed with its fatality on his going to a village near St. Peterfburgh, to inquire for matter for inoculation, and finding that of thirty-feven perfons attacked with it two only had furvived.

The

The ſecond chapter contains additional obſervations to a Treatiſe on Inoculation, formerly publiſhed by the author. He now objects leſs to inoculation in early infancy, but conſiders children with heads remarkably large as exceptionable ſubjects. In two inſtances of this ſort he has ſeen the eruptive fever attended with a ſtupor, which in one of the patients proved fatal. He now thinks preparation, and reſtriction in diet, previous to the inoculation, may be diſpenſed with. To perſons of very delicate habits, he has even allowed light animal food, with a glaſs or two of wine at dinner, during the whole time previous to the eruptive fever.

At the end of his chapter on preparation, in his former treatiſe, he has mentioned an inſtance of a child born nine weeks after inoculation, at the full time, with diſtinct marks of the diſeaſe, though the mother had very few eruptions. He has ſince ſeen inſtances in which two pregnant women were inoculated, and each had a plentiful eruption of ſmall pox: three or four years afterwards he inoculated the children, and both had a tolerable number of puſtules. Altho' of many pregnant women whom he has inoculated not one miſcarried during the diſeaſe, yet he has known miſcarriages to happen in a ſhort time after after their recovery; he therefore thinks it unadvifable to inoculate women in that ftate, unlefs the neceffity of the cafe requires it.

Cool air and evacuations, though neceffary when the eruptive fever runs high, may, in the opinion of our author, be difpenfed with when the complaints are moderate, and the patient delicate. When the eruption is completed, he thinks a very cold regimen may be hurtful. If the puftules are numerous, he advifes confinement to a chamber for eafe. He has feen a fudden tranfition from a warm, clofe room to a cool, airy one prove very dangerous to the patient. Extremes therefore are to be cautioufly avoided.

If the eruption is large; if the fever remains in any confiderable degree after the eruption, and the fkin feels ftretched and painful, but more efpecially if the throat be fore, he recommends a blifter of the fize of an Englifh crown-piece to be applied upon the very place of the arm where the incifion was made, and fuffered to remain on about twelve hours. This application, we are told, will almoft infallibly produce both fpeedy and confiderable relief; nor does it hinder the fore from healing, as fome might expect, for when both arms have been inocuinoculated, the bliftered incifion has moft commonly healed fooner than the other. When the inoculated part inflames confiderably, fo as to occafion great reftleffnefs and fever, as happens particularly in young children, a common cataplafm of bread and milk is faid to give relief.

In addition to what he had formerly faid of anomalous fymptoms, the author obferves, that fometimes a patient, who has paffed through the eruptive fever, with moderate fymptoms, has been unexpectedly attacked with reftleffnefs and an alarming degree of fever, and very frequently, in children, with uncommon fits of crying. For a time he was unable to explain thefe fymptoms, but he is now convinced they originate from puftules fituated in the internal parts of the mouth, or of the nofe or œfophagus. The great duke's cafe was of this kind. He has always treated this complaint fuccefsfully, by producing a flight perfpiration, by which means the whole difturbance has commonly been over in twenty-four hours.

In the third chapter the author treats of epidemic fmall pox. He confiders the variolous matter as a poifon, *fui generis*, the operation of which moft of the human fpecies are liable to to experience once in their lives, but very rarely twice. He does not believe that the ſtate of the air ever generates the ſmall pox, unleſs aided by contagion; neither will he allow that the ſmall pox returns epidemically at certain periods, but he believes that certain ſeaſons and conſtitutions of the air are more favourable than others to propagate the diſtemper. As proofs that it is not generated by any peculiar ſtate of the air, he obſerves, that it was unknown in America till conveyed thither by Europeans about 200 years ago; that in ſome of the northern parts of Europe it has not made its appearance above 70 or 80 years; that no traces of it were to be found in Siberia until the Ruſſians carried it thither; that ſome parts of Tartary are ſtill free from it; and that the Iſland of St. Helena remains alſo to this day uninfected, and its inhabitants, who juſtly dread the introduction of the diſeaſe, uſe every precaution to prevent it.

In his fourth chapter, the author gives ſome obſervations in favour of the opinion, that the ſmall pox attacks the ſame perſon but once. He remarks, that the diſeaſes which have been moſt frequently taken for ſmall pox are ſwine and chicken pox. Theſe two are generally ſuppoſed to be the ſame, but from a caſe related of of a child who underwent them both, they appear to be diſtinct diſorders. The Baron relates ſeveral inſtances where the matter of the ſwine or chicken pox has by miſtake been uſed in inoculation inſtead of the ſmall pox. He declares, that in a practice of more than forty-ſix years, he has not met with a ſingle inſtance of any perſons having the ſmall pox twice.

He takes occaſion to notice a remarkable fact related by Mr. Mudge, in a late publication, and which is briefly as follows: of forty perſons inoculated at Plymouth, thirty were inoculated with crude matter taken from the arm of a woman five days after ſhe herſelf had been inoculated with concocted matter; the other ten were inoculated with concocted matter of the natural ſmall pox. The arms of all the forty ſeemed to have taken the infection, yet only the latter ten had the eruptive fever and ſmall pox in the uſual way. The other thirty having been afterwards inoculated again with concocted matter had the diſeaſe in the uſual manner. Our author ſuppoſes, that in this caſe the failures aroſe from too precipitately forming an opinion, that an inflammation, and a ſore with punctured matter on the punctured part, would certainly convey the diſeaſe, without conſidering ſo accurately as was neceffary, whether fuch inflammation and fore were truly of a variolous nature, which, in the inftance juft now mentioned, he thinks was not the cafe, and confequently that the inoculation of the thirty patients having been performed from a difcharge not impregnated with the variolous poifon, the fmall pox could not be produced.

The Baron obferves, that once in his life he has fucceeded in procuring fufficient infection for inoculation fo early as the 4th day, but that in general it is very rarely to be procured even on the fifth, although on the 8th, 9th, and 10th it is common. Not a fingle inftance has occurred to him where he could have inoculated four perfons from the arm of any one patient on the fifth day, fo that, of courfe, if he had feen, as in Mr. Mudge's patient, inflammation and difcharge enough to inoculate thirty at fo early a period, he fhould have attributed it to fome conftitutional appearance, and have thought it unfit for conveying the fmall pox. He is ftill perfuaded, that provided due care has been taken to procure infection from true fmall pox, and the progrefs in confequence of it, in the inoculated perfon, has been agreeable to what is ufually obferved in inoculated patients, the crude crude matter will be equally efficacious with that which has been more concocted. He obſerves, that if the inoculation be performed with fluid matter, no conſtitution of the air or body can reſiſt its operation; but that if the matter is diluted with a ſmall proportion of water, it is uſually ſlower in producing the diſeaſe.

In the following chapter the author endeavours to prove that ſome perſons paſs through life without appearing to be capable of receiving the ſmall pox. That every perſon is liable to receive this infection once, has been a generally received opinion; but as ſome perſons have paſſed thro' a long life, without taking the diſeaſe, although frequently in the way of infection; and as others have been repeatedly inoculated, and afterwards intentionally expoſed to the worſt kind of natural ſmall pox, without any appearance of diſeaſe being produced, the concluſion will be warranted, that ſome are ſo conſtitutionally framed, as not to be ſubject to this diſtemper in any degree.

Inſtances of adults, upon whom repeated inoculations have produced no effect, would not readily be admitted as proofs of this poſition; for to ſuch it might be objected, that they had paſſed through the diſeaſe in a ſlight manner in ſome early period of their lives. Baron Dimſdale therefore offers no other evidence than that of infants. He relates three inſtances of children, in the earlieſt ſtate of infancy, who ſhewed not the leaſt ſigns of receiving the variolous infection, though repeatedly inoculated and expoſed to the natural ſmall pox.

In the ſixth chapter our author communicates an important and intereſting diſcovery, which is, that although a perſon, who has never had the ſmall pox, has continued to reſide in an infected apartment ſo long, that there muſt be the higheſt probability of the infection being taken, yet even under theſe alarming circumſtances, if inoculation is performed, he ſhall have the diſtemper from the inoculation only, with every advantage ariſing from it; and the natural infection ſhall be, as it were, ſuperſeded. Several caſes are related in proof of this poſition. Baron Dimſdale ſuppoſes the cauſe of this ſingular circumſtance is, that the natural and inoculated diſeaſes take place after different periods of infection. It is well known, that inoculated patients uſually begin to complain on the 8th, 9th, or 10th day; the exact time of receiving the natural infection is unknown, yet from accounts counts well authenticated, and from his own experience, our author is inclined to think that from 13 to 20 days, or more, ufually intervene before the commencement of the eruptive fever.

Inoculation fometimes failed when practifed in the old manner; this might be owing to the dry ftate of the matter inferted, and the dreffings applied over it; whereas in the prefent method, the matter is generally frefh, or if a few days old, and dry, it is moiftened by fteam, or with a little warm water juft fufficient for the purpofe of dilution; and therefore Baron Dimfdale maintains, that the fuccefs of this new method is infallible, provided the patient is capable of receiving infection, fo that if no infection appears on the firft trial, no future inoculation will fucceed.

Dr. Archer, phyfician to the Small Pox Hofpital, has obferved, that perfons fuppofed to have the fmall pox are frequently fent there, in whom the eruption proves to be of fome other kind. In fuch cafes compaffion to patients brought into a houfe loaded with infection induces him to fuffer them to remain there until the real difeafe appears; and he finds that patients thus circumftanced, almoft conftantly have the fmall pox in a favourable manner. Our author afcribes this to their receiving the infection by cutaneous abforption, as every part of the furniture is certainly in a high ftate of infection, and the infection by contact, as well as by inoculation, being quicker in its operation, fuperfedes the progrefs of infection taken in any other manner.

The feventh and laft chapter contains conjectures on the probable caufes of the different kinds and degrees of natural fmall pox, and on the different fuccefs of the methods adopted in the practice of Inoculation.

The author fets out with obferving, that but little dependence can be placed on good health and regularity of diet as a fecurity againft a dangerous kind of fmall pox, the moft luxurious and irregular livers being frequently obferved to have it in the mildeft manner, while children in early infancy, and delicate and abftemious females, who might be fuppofed to be in a perpetual ftate of preparation, often fall victims to its malignity. He therefore fuppofes, that fome are fo conftitutionally formed as to receive the fmall pox in a milder or more violent degree.

When the poifon of the fmall pox is abforbed, as in inoculation, he fuppofes, that the quantity of poifon generated is fmall; that the organs firft affected are not of the vital kind, and and that in these, probably, part of the virulence is exhausted; whereas in bad cases of natural small pox a greater quantity of the infection has been received internally, and produces an universal and dangerous disease.—From a dissection at which he was present some years ago, at St. Thomas's Hospital, he is of opinion, that in bad confluent kinds the internal parts are covered with pustules.

Towards the close of the work the author points out the inconveniences of the old method contrasted with the advantages of the new one; "of which," says he, with great candour, "I "shall now, as indeed I have done at all times "and on every occasion, give the whole merit "to the family of the SUTTONS." The essential difference, he observes, consisted in returning to the original method of a slight puncture and the use of recent fluid matter, without applying a dressing of any kind to the part, and enjoining the use of cold air and cold drink. In the old practice a greater quantity of matter was used, and as a large ulcerated surface was produced, the absorption would of course be greater. The present method is free from this inconvenience.

It may be suggested perhaps by some readers, that the author has not given due credit to the ininfluence of the open air and cold water. He acknowledges their utility in *some* cases, but he is far from being convinced of their efficacy in every case. His practice in Russia undeceived him on this head, for during the severity of a Russian winter it would have been an unpardonable rashness to have ordered patients into the open air, or indeed to have enjoined them to reside in unwarmed apartments; so that he directed the regulation of the rooms to a temperate heat by the thermometer, and this method succeeded to his satisfaction.

We have thus given a summary view of a performance which we have perused with singular satisfaction. Exclusive of many pleasing anecdotes, related in a manner that does honour to the candour and good sense of the noble author, it abounds with new and important observations, which cannot but render it a valuable acquisition to the medical reader.

